# Decision-making using absolute cardiovascular risk reduction and incremental cost-effectiveness ratios: a case study

**Published:** 2008-04

**Authors:** J A Ker, H Oosthuizen, P Rheeder

**Affiliations:** Department of Internal Medicine, School of Medicine, Faculty of Health Sciences, University of Pretoria, Pretoria; Medical Advisor, sanofi-aventis; Clinical Epidemiology Unit, School of Medicine, Faculty of Health Sciences, University of Pretoria and Pretoria Academic Hospital, Pretoria

## Abstract

**Background:**

Many clinical guidelines have adopted a multifactorial cardiovascular risk assessment to identify high-risk individuals for treatment. The Framingham risk chart is a widely used risk engine to calculate the absolute cardiovascular risk of an individual. Cost-effective analyses are typically used to evaluate therapeutic strategies, but it is more problematic for a clinician when faced with alternative therapeutic strategies to calculate cost effectiveness.

**Aim:**

We used a single simulated-patient model to explore the effect of different drug treatments on the calculated absolute cardiovascular risk.

**Methods:**

The Framingham risk score was calculated on a hypothetical patient, and drug treatment was initiated. After every drug introduced, the score was recalculated. Single-exit pricing of the various drugs in South Africa was used to calculate the cost of reducing predicted cardiovascular risk.

**Results:**

The cost-effective ratio of an antihypertensive treatment strategy was calculated to be R21.35 per percentage of risk reduction. That of a statin treatment strategy was R22.93 per percentage of risk reduction. Using a high-dose statin, the cost-effective ratio was R12.81 per percentage of risk reduction. Combining the antihypertensive and statin strategy demonstrated a cost-effective ratio of R23.84 per percentage of risk reduction. A combination of several drugs enabled the hypothetical patient to reduce the risk to 14% at a cost-effective ratio of R17.18 per percentage of risk reduction.

**Conclusion:**

This model demonstrates a method to compare different therapeutic strategies to reduce cardiovascular risk with their cost-effective ratios.

## Summary

The cardiovascular disease burden in South Africa is high and, based on a recent report of the Medical Research Council, hypertension ranks as the second highest cause of death in South Africa, following infectious causes.^1^ Death rates from obesity, high cholesterol and diabetes were respectively ranked five, seven and eight in importance.^1^ There is also a high prevalence of risk factors such as hypertension, diabetes and the metabolic syndrome in black South African patients with coronary artery disease, as was recently described.^2^

Cardiovascular disease remains a major cause of disability and death around the globe. Treatment aimed at modifiable risk factors such as hypertension, hypercholesterolaemia and smoking can delay or reduce the risk of developing cardiovascular disease.^3^-^5^ Making correct decisions on optimal treatment is essential for both the clinical healthcare provider as well as the funder of often expensive and multiple interventions addressing cardiac risk.

In the past, patients were treated based on the presence or absence of an individual cardiovascular risk factor, an approach that appears straightforward, but may have resulted in some individuals receiving unnecessary treatment that may not have delivered the desired outcome or, alternatively, not treating individuals at high risk.^6^ Adopting a multifactorial cardiovascular risk-assessment approach to identify high-risk individuals who need interventions has been introduced into some clinical guidelines, whereby the initiation of therapy is based on the predicted absolute cardiovascular risk of the individual.^7^,^8^

Two of the most widely used risk ‘engines’ to calculate absolute cardiovascular risk are the Score project in Europe and the Framingham risk chart of the USA.^8^,^9^ No single risk calculator can claim to be the perfect instrument and all have limitations. Furthermore, multiple risk-factors interventions in identified high-risk patients are more beneficial than single risk-factor interventions.^10^ After initial therapy aimed at reducing absolute cardiovascular risk, subsequent therapy could be tailored for additional risk reduction according to expected benefit and cost.

The latter approach may be of particular importance for the healthcare funder who needs to make re-imbursement decisions regarding cardiovascular risk management. It may be useful to establish a framework by which treatments are chosen because of their cost effectiveness, based on changes in cardiovascular risk and drug costs.

The results of a cost-effectiveness analysis are typically reported as cost effectiveness ratios where treatment costs comprise the numerator and the effectiveness measure serves as denominator. Average cost-effectiveness ratios, however, have limited value when deciding on alternative therapeutic strategies, where incremental cost-effectiveness ratios (ICER) may be more beneficial.^11^ For example, cost of drug A is R100.00, outcome is 5% reduction in risk (from an arbitrary baseline); drug B is R200.00, outcome is 15% risk reduction (from the same baseline). ICER = (R200–R100)/(15%–5%) = R100/10% = R10/% risk reduction. This is the incremental cost effectiveness when switching from drug A to drug B. The average cost effectiveness of drug B would be R200/15% = R13.33/% risk reduction. Drug C costs R300 with an 18% reduction in risk. Switching from drug A means the ICER = (R300–R100)/(18%–5%) = R200/13% = R15.38/% risk reduction. One may then choose drug B rather than C based on incremental cost-effectiveness.

With these background facts, our aim was to use a single simulated-patient model to explore the effect of drug treatment on the calculated absolute cardiovascular risk and the possible cost implications of using such an approach in decision-making.

## Methods

The hypothetical case study used to perform the calculations was a male smoker, 56 years old, blood pressure 160/100 mmHg, total cholesterol 6 mmol/l, low-density lipoprotein (LDL) cholesterol 4.2 mmol/l, and high-density lipoprotein (HDL) cholesterol 0.7 mmol/l. Our hypothetical case study was free of cardiovascular disease. The Framingham risk chart we used to determine the absolute risk of this hypothetical patient to develop a coronary heart disease (CHD) event over 10 years was calculated to be 40% (high risk).^9^ We used the Framingham risk score, as published by Wilson *et al.*,^9^ with age as step 1, LDL cholesterol as step 2, HDL cholesterol as step 3, blood pressure as step 4, presence or absence of diabetes as step 5, and smoking status as step 6.

The coronary heart disease risk for a person of the same age at average risk would be 16%, and for a person of the same age at low risk to develop a coronary heart disease event would be 7% using the same table.^9^ The relative risk of our case compared to a similar-aged, low-risk patient would be 40/7 = 5.7%. Target blood pressure according to the South African guidelines should be < 140/90 mmHg.^12^ His total cholesterol should be < 5.0 mmol/l (desirable level), LDL cholesterol ≤ 2.6 mmol/l, HDL cholesterol ≥ 1.0 mmol/l and he should quit smoking.^13^ If these targets were met, his CHD risk according to the Framingham table calculator would then be 11% and his relative risk compared to a similar-aged, low-risk male would be 1.57%, which is still at an increased risk but lower than his initial relative risk of 5.7%.

To mimic the risk reduction following implementation of therapy in this patient, his cardiovascular risk was recalculated every time a new drug was introduced. The effect of quitting smoking was calculated once only to demonstrate the effect on the absolute risk, but was not taken into consideration again when drugs were added, as we did not calculate the cost of smoking cessation.

There is an extensive range of medication available for the management of cardiovascular risk factors. To simplify the approach of this model, the choice of medication used on the patient in this model was based on the most commonly prescribed medication in South Africa in a specific class according to IMS data (June 2006), reflecting the prescribing habits of healthcare providers in South Africa. We supposed that current prescribing practices may better reflect reality. We did not explore the effect of classes of drugs on the outcome of this model. The prices quoted reflect the single-exit pricing (SEP) at the end of 2006. We used randomised clinical trials or meta-analyses to obtain efficacy estimates. These only serve as examples as no systematic review regarding efficacy was done.

## Results

If the patient quit smoking, his absolute risk would decrease by 13% from 40% to 27%, his relative risk compared to an agematched, low-risk male would be 27/7 5 3.86%. No cost was included in these calculations.

The antihypertensive medication used, perindopril-indapamide combination (4 mg/2.5 mg), has a blood pressure-lowering effect of 12.3/5 mmHg based on the Progress trial.^14^ The absolute coronary heart disease risk would then change to 33% (7% reduced), from baseline 40%, at a cost of R149.46 per month. The average cost-effectiveness ratio would be R149.46/7% = R21.35/% risk reduction.

We can choose a strategy to first treat his dyslipidaemia before later treating his blood pressure by starting on a statin. Atorvastatin 10 mg, based on the Stellar trial,^15^ lowers total cholesterol by 27.1% from 6.0 mmol/l to 4.37 mmol/l, lowers LDL cholesterol by 36.8%, from 4.2 mmol/l to 2.66 mmol/l and increases HDL cholesterol by 5.7% to 0.74 mmol/l, at a cost of R160.50 per month. His absolute cardiovascular risk would be reduced by 7% to 33%. The average cost-effectiveness ratio would be R160.50/7% = R22.93/% risk reduction.

Yet another strategy would be to initiate atorvastatin 40 mg, at a cost of R281.78 per month. The total cholesterol would be lowered by 35.8% from 6.0 mmol/l to 3.85 mmol/l, the LDL cholesterol would be lowered by 47.8% to 2.19 mmol/l and the HDL cholesterol would be increased by 4.4% to 0.73 mmol/l. This would reduce his global cardiovascular risk to 18%. The average cost-effectiveness ratio would be R281.78/22% = R12.81/% risk reduction. The incremental cost-effectiveness ratio would then be R8.82 per additional % risk reduction compared to hypertension treatment alone.

Another strategy would be to initiate a combination treatment of perindopril-indapamide with atorvastatin 10 mg. Then the absolute CHD risk would be reduced to 27%. The total cost of the combination of drugs used is R309.96 per month and the average cost-effectiveness ratio then would be R309.96/13% = R23.84/% risk reduction. These results as well as the incremental cost-effectiveness ratios are shown in Table 1.

**Table 1.** Characteristics Of Patient And Different Treatment Strategies

	*Baseline*	*Quit smoking only*	*HT treatment*	*Lipid treatment 10 mg*	*Lipid treatment 40 mg*	*HT and lipid treatment (10 mg) (Combination A)*
Age	56	56	56	56	56	56
Gender	M	M	M	M	M	M
Smoker	+	-	+	+	+	+
SBP (mmHg)	160	160	148	160	160	148
DBP (mmHg)	100	100	95	100	100	95
TC (mmol/l)	6.0	6.0	6.0	4.37	3.85	4.37
LDL-C (mmol/l)	4.2	4.2	4.2	2.66	2.19	2.66
HDL-C (mmol/l)	0.70	0.70	0.70	0.74	0.73	0.74
Framingham absolute risk (%)	40	27	33	33	18	27
Relative risk vs low-risk patient	5.7	3.85	4.71	4.71	2.57	3.86
Monthly cost			R149.46	R160.50	R281.78	R309.96
ACER (Rand / % risk reduction)			R21.35	R22.93	R12.77	R23.84
ICER (Rand / % risk reduction)				Hypertension treatment dominant	R8.77 compared to HT treatment	R26.75 compared to HT treatment. R24.91 compared to 10-mg lipid treatment

HT: hypertension; SBP: systolic blood pressure, DBP: diastolic blood pressure; TC: total cholesterol; LDL-C: low-density lipoprotein cholesterol; HDL-C: high-density lipoprotein cholesterol; ACER: average cost-effectiveness ratio; ICER: incremental cost-effectiveness ratio.

After initiating the combination of perindopril–indapamide–atorvastatin, the clinician who wishes to reduce the risk further may now either lower the blood pressure or the cholesterol further. Adding a calcium channel blocker, amlodipine 5 mg (the most commonly prescribed calcium channel blocker in South Africa) to the perindopril–indapamide combination is one option. The effect of amlodipine 5 mg as monotherapy would be a lowering of 10.3/10.1 mmHg for the systolic and diastolic blood pressure, respectively.^16^ If these values are used addition ally to those of the initial blood pressure lowering, the newly calculated blood pressure would be 137.4/84.9 (138/85) mmHg. The CHD risk would then be lowered to 22% if amlodipine was added to the atorvastatin–perindopril combination. If we used all these drugs in combination, the average cost-effectiveness ratio would be R412.97/18% = R22.94/% risk reduction. The incremental cost-effectiveness ratio of a strategy of adding amlodipine to the combination of perindopril–indapamide and atorvastatin (Combination A) would be R412.97–R309.96/18–13% = R20.60/additional % risk reduction (Table 2).

**Table 2.** Different Drug Combination Strategies

	*Combination A: add amlodipine*	*Combination A: add fibrate*	*Combination B: all drugs combined*
Age	56	56	56
Gender	M	M	M
Smoker	+	+	+
SBP (mmHg)	134.5	147.7	134.5
DBP (mmHg)	87.1	95	87.1
TC (mmol/l)	4.37	3.65	3.65
LDL-C (mmol/l)	2.66	2.31	2.31
HDL (mmol/l)	0.74	0.82	0.82
Framingham absolute risk (%)	22	14	11
Relative risk vs low-risk patient	3.14	2.0	1.57
Monthly cost	R412.97	R446.75	R546.76
ACER (Rand / % risk reduction)	R22.94	R17.18	R18.85
ICER (Rand / % risk reduction)	R20.60 compared to Combination A alone	R10.52 compared to Combination A alone	R14.80 compared to Combination A

SBP: systolic blood pressure, DBP: diastolic blood pressure; TC: total cholesterol; LDL-C: low-density lipoprotein cholesterol; HDL-C: high-density lipoprotein cholesterol; ACER: average cost-effectiveness ratio; ICER: incremental cost-effectiveness ratio.

An alternative strategy would be not to add amlodipine but to add a fibrate to further correct the lipid profile. According to the IMS data, the most commonly prescribed fibrate is bezafibrate. Adding bezafibrate would increase the HDL cholesterol by 11% to 0.82 mmol/l, decrease total cholesterol by 10%, and LDL cholesterol by 13%, based on a meta-analysis. 17 This would change the patient’s risk profile to 14%. The average cost-effectiveness ratio of this combination strategy R446.75/26% 5 R17.18 / % risk reduction. The ICER moving from a strategy without bezafibrate to one with bezafibrate = R446.75–R309.96/26–13% = R10.52/additional % risk reduction (Table 2).

If all the medications are used in combination: the ACE–diuretic combination, atorvastatin (10 mg), amlodipine and bezafibrate, the absolute risk of the hypothetical case would be 11%, at a monthly cost of R546.76. The average cost-effectiveness ratio would then be R18.85/% risk reduction. The incremental cost-effectiveness ratio of this combination (Combination B), compared to the initial combination of atorvastatin (10 mg)–perindopril–indapamide (Combination A) would be R14.80 per additional % risk reduction (Table 2).

## Discussion

We used a single simulated patient at high calculated risk to develop coronary heart disease to demonstrate the effect of various drugs on this risk and to calculate the cost effectiveness based on predicted risk reduction.

In our simulated single-patient model, the CHD risk could be reduced to 27% by a strategy of quitting smoking, although it was difficult to predict the real effect on risk, or a strategy of using a combination of two antihypertensive drugs together with a low-dose statin. The average cost-effectiveness ratio for treating the patient with a high dose of statin appears to be the most effective strategy for reducing risk.

In a previous study, using a simulated population and different risk calculators for the same person, the authors found that it could lead to different results and different economic consequences. 18 We used a single simulated patient and a single risk table to demonstrate the effect of medication with cost implications on reducing calculated absolute cardiovascular risk.

In our simulated patient, the calculated absolute CHD risk was 45% over 10 years, which is a much higher threshold to initiate treatment than the suggested arbitrary threshold for initiation of treatment of 20%. In identifying potential modifiable risk, which is the maximum reduction in absolute risk, an ideal patient with low risk, the systolic blood pressure would be 120 mmHg, total cholesterol-to-HDL cholesterol ratio would be 4 and such a patient would not smoke. If a level of 20% absolute risk over 10 years is used to initiate treatment, it would lead to exclusion of a large group of relatively young patients who would benefit from treatment.^19^

We did not calculate the cost or the necessary combination of drugs to eliminate all modifiable risk, as was done previously by other authors.^19^ We calculated the cost effect of reducing the absolute risk in our model to 11%, which was as close as possible to 7%, the absolute risk of a low-probability risk category patient in the Framingham table. The problem with aiming for a specific level of risk using a level of 7%, as we did, is the absence of clinical trial and cohort study data to define the risk, benefits and cost of interventions based on global risk, and the fact that the thresholds for initiating therapy based on global cardiovascular risk are arbitrary.^20^ To what level should the risk be reduced? We could only reduce the risk of our patient to 11%.

In a recent study, researchers used these principles to demonstrate the effect of weight loss with bariatric surgery on the predicted coronary heart disease risk.^21^ They quantified the global decrease in coronary heart disease risk due to sustained weight loss using a similar method to the one we used.

The cost advantages of the use of absolute risk assessment in the management of patients still need to be clearly established. Our model serves as one method to measure cost advantages, as it can be used to calculate the cost in Rand per percentage reduction in the absolute risk of the patient. This model enables one to compare different drugs and may also be a method to calculate the efficiency of a new drug entering the market.

As in any model, there are limitations and the same limitations apply to our model. The efficacy data used for blood pressure lowering with the perindopril–indapamide combination may differ as separate entities from that of a fixed-combination pill. Fixed combinations of cardiovascular drugs may have synergistic effects that cannot be accounted for in such a theoretical model. Another limitation is the estimation of the effect of blood pressure lowering with amlodipine as add-on medication to an ACE–diuretic combination, as no publication with this information could be found.

In the South African perspective, the different ethnic groups may also have different results when evaluating cardiovascular risk, as the risk calculators are mainly based on Caucasian patients. Even though these risk models have not been validated in South Africa, the absolute effect on risk-factor reduction achieved by treatment would still most likely be relevant, especially in the Caucasian population.

The effect of smoking can be quite complex and difficult to apply in risk calculators. If our hypothetical patient quit smoking, the smoking cessation would not put our patient at the same risk level as a non-smoker and the pack-years of exposure would also contribute to the cardiovascular risk.

These calculations are based on a hypothetical case study and may differ if used to evaluate a population. As noted before, population simulation studies may provide estimates as well as confidence intervals for the incremental cost-effectiveness ratios. The confidence intervals may make the predictions more precise.

The cost advantages of the use of comprehensive risk assessment, as used in our case study in the management of patients has yet to be established and the costs may be higher than anticipated.^18^ There will be cost differences between lowering the absolute risk to below 20% versus lowering the risk to that of a similar low-risk individual (7% in the case of Framingham tables) or versus a strategy to eliminate all modifiable risk, as was done previously.^19^

Medication side effects were not included in any of these calculations. The drug side-effect profile may also influence compliance of patients and thereby have an effect on the outcome data. We also did not attempt to put a cost to reduction in event outcomes, such as myocardial infarctions in this model.

In conclusion, this simulated case demonstrates the variations and limitations of using risk calculators to decide on therapy or not. Various therapeutic options could be explored with incremental cost-effectiveness ratios using this model, but the limitations of risk calculations should be borne in mind.
